# Prophylactic Effects of *Bifidobacterium adolescentis* on Anxiety and Depression-Like Phenotypes After Chronic Stress: A Role of the Gut Microbiota-Inflammation Axis

**DOI:** 10.3389/fnbeh.2019.00126

**Published:** 2019-06-18

**Authors:** Ying Guo, Jian-Ping Xie, Ke Deng, Xia Li, Yun Yuan, Qun Xuan, Jing Xie, Xiao-Ming He, Qian Wang, Juan-Juan Li, Huai-Rong Luo

**Affiliations:** ^1^State Key Laboratory of Phytochemistry and Plant Resources in West China, Yunnan Key Laboratory of Natural Medicinal Chemistry, Kunming Institute of Botany, Chinese Academy of Sciences, Kunming, China; ^2^Key Laboratory for Aging and Regenerative Medicine, Department of Pharmacology, School of Pharmacy, Southwest Medical University, Luzhou, China; ^3^School of Basic Medical Sciences, Kunming Medical University, Kunming, China; ^4^University of Chinese Academy of Sciences, Beijing, China; ^5^Library, Yunnan Minzu University, Kunming, China

**Keywords:** *Bifidobacterium adolescentis*, antidepressant, chronic restraint stress, gut microbiota, inflammation

## Abstract

Stress disturbs the balance of the gut microbiota and stimulates inflammation-to-brain mechanisms. Moreover, stress leads to anxiety and depressive disorders. *Bifidobacterium adolescentis* displays distinct anti-inflammatory effects. However, no report has focused on the anxiolytic and antidepressant effects of *B. adolescentis* related to the gut microbiome and the inflammation on chronic restraint stress (CRS) in mice. We found that pretreatment with *B. adolescentis* increased the time spent in the center of the open field apparatus, increased the percentage of entries into the open arms of the elevated plus-maze (EPM) and the percentage of time spent in the open arms of the EPM, and decreased the immobility duration in the tail suspension test as well as the forced swimming test (FST). Moreover, *B. adolescentis* increased the sequence proportion of *Lactobacillus* and reduced the sequence proportion of *Bacteroides* in feces. Furthermore, *B. adolescentis* markedly reduced the protein expression of interleukin-1β (IL-1β), tumor necrosis factor α (TNF-α), p-nuclear factor-kappa B (NF-κB) p65 and Iba1 and elevated brain derived neurotrophic factor (BDNF) expression in the hippocampus. We conclude that the anxiolytic and antidepressant effects of *B. adolescentis* are related to reducing inflammatory cytokines and rebalancing the gut microbiota.

## Introduction

With high mortality and morbidity, depression is a common and recurrent mood disorder accompanied by behavioral deficits (McKeever et al., 2017). Evidence shows that response to the first-line treatment of depression is 40%–60%, while remission following antidepressant treatment is 30%–45% (Trivedi et al., 2006). Thus, our efforts have focused on developing better antidepressant drugs.

In recent years, emerging evidence has suggested an involvement of the gut microbiota in inflammation, brain development and behavior (Evrensel and Ceylan, 2015). The relationship between microbiota and anxiety/depression has been studied by the chronic restraint stress (CRS) model, in which the gut microbiota, specifically the abundance of *Allobaculum*, *Bifidobacterium*, *Turicibacter* and *Clostridium*, is altered (Wong et al., 2016). It is becoming apparent that probiotics induce substantial impacts on the health of the host. The absence of probiotic bacteria in the gut is implicated in the etiology of depression (Desbonnet et al., 2008), and additionally, the prolonged intake of probiotics (*Lactobacillus helveticus* R0052 and *Bifidobacterium longum* R0175) has favorable effects on anxiety- and depression-related behaviors without the presence of any adverse events (Messaoudi et al., 2011). Studies have found that *Bifidobacterium* has positive effects on stress-related diseases (Meyer and Vassar, 2018), and furthermore, the probiotic *Bifidobacterium infantis* may possess antidepressant properties (Desbonnet et al., 2008). However, the exact mechanisms underlying the antidepressant effect of *Bifidobacterium* in connection with the brain-gut axis remain poorly understood.

Inflammatory mechanisms mediate increased stress responsiveness and depression susceptibility (Hennessy et al., 2019). The increased release of peripheral cytokines exacerbates anxiety- and depression-like behaviors in stressed animals (Hodes et al., 2014). In addition, interleukin (IL)-1β is pivotal to the acquisition of depressive phenotypes in stressed animals (Maes et al., 2012). Antidepressant effects occur when the interleukin-1β (IL-1β) level is decreased (Zhang et al., 2015). The data from a clinical study suggest that plasma tumor necrosis factor (TNF)-α is also correlated with depression severity (Oglodek et al., 2017), and anti-TNF-α treatment alleviates depressive mood (Krishnan et al., 2007). In chronic stress models, microglial activation is significantly increased in the cingulate and medial orbital cortices, nucleus accumbens, caudate putamen, amygdala and hippocampus in the mouse brain (Farooq et al., 2012), and furthermore, the levels of IL-1β, IL-6 and tumor necrosis factor α (TNF-α) are increased in the substantia nigra (de Pablos et al., 2014). Stress can disturb the balance of the gut microbiota, stimulate inflammation-to-brain mechanisms, and lead to microglia activation in depressive disorders (Maes, 2008). Nuclear factor κB (NF-κB) is one of the major transcription factors that mediate inflammatory responses, and the activation of NF-κB in HT-29 cells can be inhibited by pre-incubation with *Bifidobacterium adolescentis* NCC251 (Riedel et al., 2006). *B. adolescentis* IM38 can regulate the *Proteobacteria* to *Bacteroidetes* ratio in the gut microbiota and inhibit NF-κB activation in the colon (Lim and Kim, 2017). *B. adolescentis* NK98 can suppress the occurrence and development of anxiety/depression, the infiltration of activated microglia into the hippocampus, and hippocampal NF-κB activation caused by acute immobilization stress (Jang et al., 2019). However, for CRS, the underlying connection between the anxiolytic and antidepressant effects of *B. adolescentis*, the gut microbiota, and inflammation are still unclear.

In this study, we investigated the anxiolytic and antidepressant effects of *B. adolescentis* related to the gut microbiota, inflammation, and behavior in CRS mice to evaluate the gut microbiota and inflammation as potential therapeutic targets in anxiety and depression.

## Materials and Methods

### Animals

Male ICR mice (6 weeks old) were purchased from Kunming Medical University. The mice were housed in groups of 4–5 per cage (290 mm × 178 mm × 150 mm) in a room under normal conditions (22 ± 1°C, 50 ± 2% humidity, and a 12-h light/12-h dark cycle) with free access to food and water. The animals were adapted to the laboratory conditions for 1 week before the experiment. The procedures were approved by the Institutional Animal Care and Use Committee of Kunming Medical University and were performed in accordance with the Guide for the Care and Use of Laboratory Animals.

### Experimental Design and Sample Collection

#### Experiment 1

**Figure 1:**
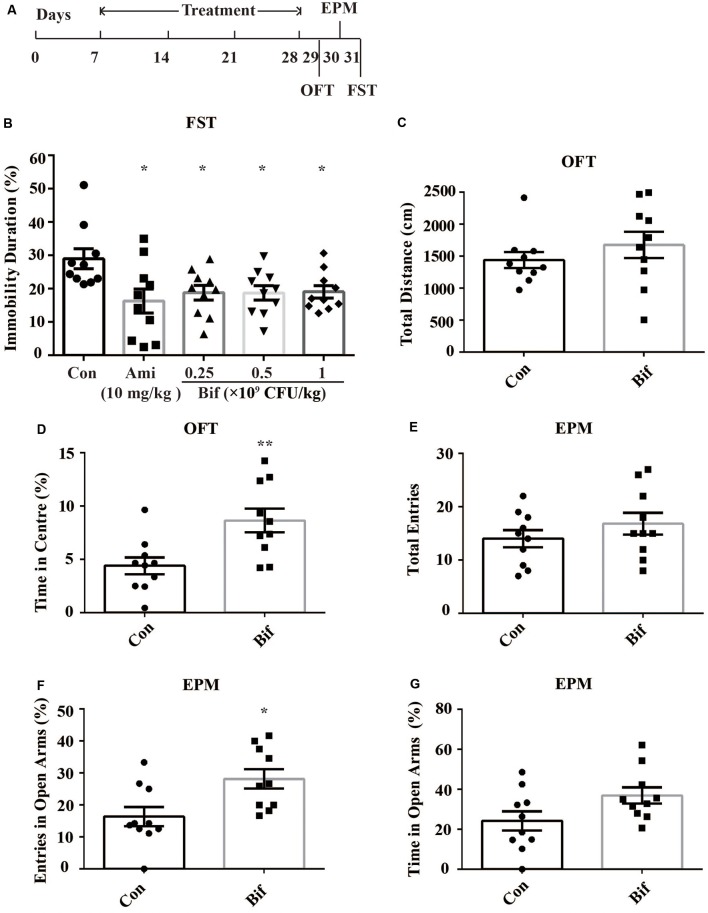
Potential anxiolytic and antidepressant effects of *B. adolescentis*. The mice in the Con, Ami (10 mg/kg), Bif (0.25 × 10^9^ CFU/kg), Bif (0.5 × 10^9^ CFU/kg), and Bif (1 × 10^9^ CFU/kg) groups were treated with 10 mL/kg distilled water, 10 mg/kg amitriptyline, 0.25 × 10^9^ CFU/kg *B. adolescentis*, 0.5 × 10^9^ CFU/kg *B. adolescentis*, and 1 × 10^9^ CFU/kg *B. adolescentis*, respectively, by gavage for 21 days. **(A)** The timeline of Experiment 1. **(B)** The results of the FST showed that the immobility duration was significantly decreased in the Ami (10 mg/kg), Bif (0.25 × 10^9^ CFU/kg), Bif (0.5 × 10^9^ CFU/kg), and Bif (1 × 10^9^ CFU/kg) groups. **(C)** The OFT showed no significant difference between the control group and the Bif (0.25 × 10^9^ CFU/kg) group in the total distance traveled. **(D)** The OFT showed that the time spent in the center was significantly increased in the Bif (0.25 × 10^9^ CFU/kg) group. **(E)** The EPM test showed no significant difference between the control group and the Bif (0.25 × 10^9^ CFU/kg) group in the total number of entries. **(F)** The number of entries into the open arms of the EPM was significantly increased in the Bif (0.25 × 10^9^ CFU/kg) group. **(G)** The EPM test showed no significant difference between the control group and the Bif (0.25 × 10^9^ CFU/kg) group in time spent in the open arms. The data are shown as the mean ± SEM. Student’s *t*-test was used. *p < 0.05 and **p < 0.01 vs. control. Con, Control; Bif, *B. adolescentis*; FST, forced swimming test; OFT, open field test; EPM, elevated plus-maze.

The mice were randomly divided into five groups: the Con group, which received 10 mL/kg distilled water; the Ami group, which received 10 mg/kg amitriptyline (the dose was determined by our preliminary experiments and the references; Manning et al., 2014; Sanna et al., 2017); the Bif 0.25 (Bif) group, which received 0.25 × 10^9^ CFU/kg *B. adolescentis*; the Bif 0.5 group, which received 0.5 × 10^9^ CFU/kg *B. adolescentis*; and the Bif 1 group, which received 1 × 10^9^ CFU/kg *B. adolescentis*. Each experimental group consisted of ten mice. The mice were treated with distilled water, amitriptyline (dissolved in distilled water), or *B. adolescentis* (dissolved in distilled water) by gavage for 21 days depending on the group. After 21 days, all mice underwent a behavioral test for three consecutive days (Figure 1).

#### Experiment 2

**Figure 2:**
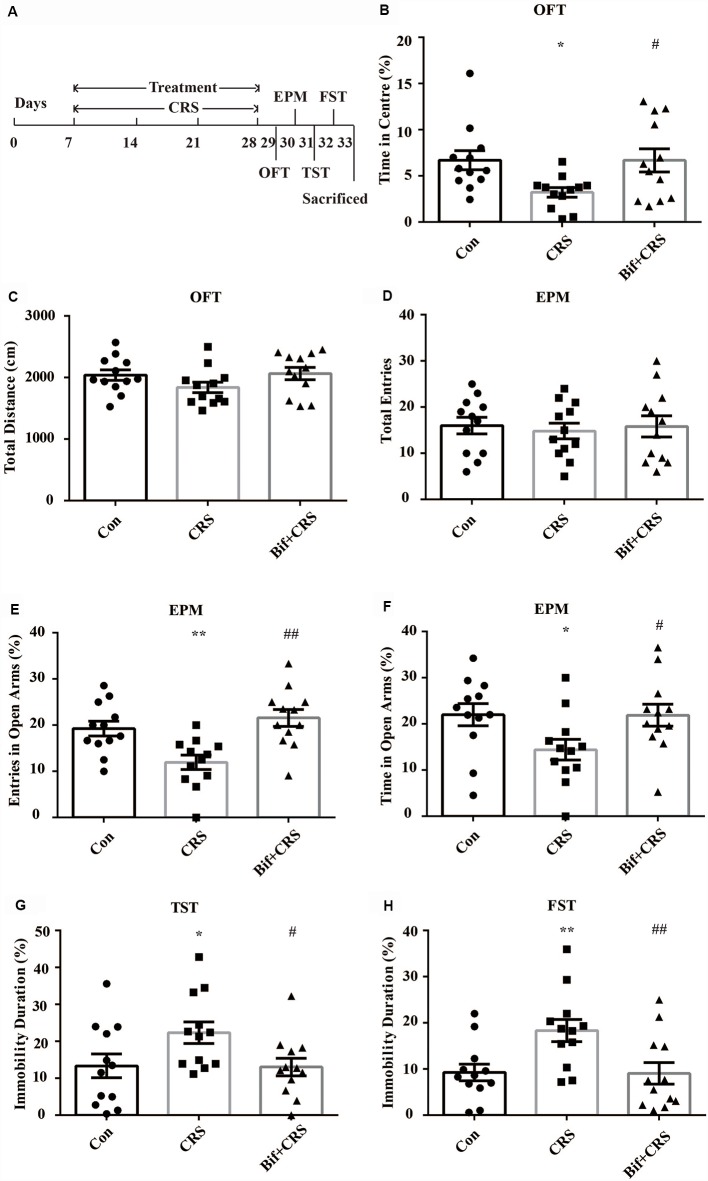
Anxiolytic and antidepressant effects of *B. adolescentis* in chronic restraint stress (CRS) mice. The mice in the Con, CRS, and Bif+CRS groups were treated with 10 mL/kg distilled water, 10 mL/kg distilled water, and 0.25 × 10^9^ CFU/kg *B. adolescentis*, respectively, by gavage for 21 days. **(A)** The schedules show the establishment of the CRS model, treatment, and behavioral tests. **(B)** The OFT showed that the time spent in the center was significantly decreased in the CRS group, and the change in the time spent in the center induced by CRS was reversed by *B. adolescentis*. **(C)** The OFT showed no significant difference among the three groups in the total distance traveled. **(D)** The EPM test showed no significant difference in the total number of entries induced by *B. adolescentis*. **(E)** The number of entries into the open arms of the EPM was significantly increased in the *B. adolescentis* group. **(F)** The time spent in the open arms of the EPM was significantly increased in the *B. adolescentis* group. **(G)** The TST showed that the immobility duration was significantly increased in the CRS group; the change in the immobility duration induced by CRS was reversed by *B. adolescentis*. **(H)** The FST showed that the immobility duration was significantly increased in the CRS group; the change in the immobility duration induced by CRS was reversed by *B. adolescentis*. The data are shown as the mean ± SEM. One-way analysis of variance (ANOVA) followed by the Student-Newman-Keuls test was used. *p < 0.05 and **p < 0.01 vs. the control; ^#^*p* < 0.05 and ^##^*p* < 0.01 vs. the CRS group; *n* = 12 per group. Con, Control; CRS, chronic restraint stress; Bif+CRS, *B. adolescentis* + chronic restraint stress; OFT, open field test; EPM, elevated plus-maze; TST, tail suspension test; FST, forced swimming test.

**Figure 3:**
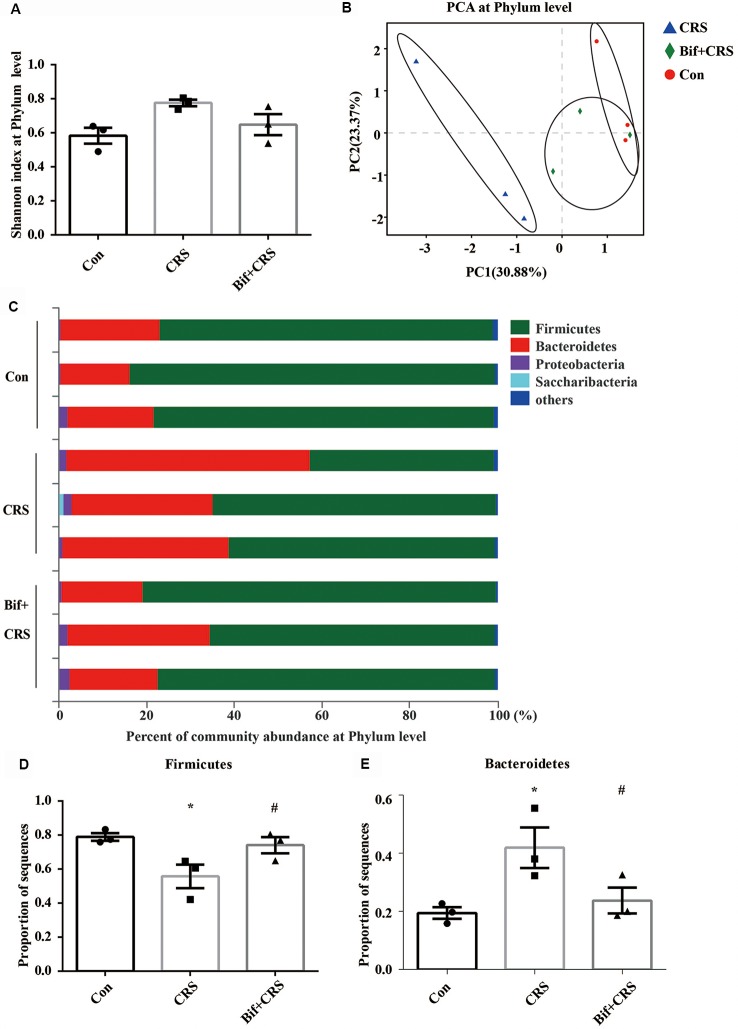
*B. adolescentis* reversed the imbalance of the intestinal microflora induced by CRS at the phylum level. The mice in the Con, CRS, and Bif+CRS groups were given 10 mL/kg distilled water, 10 mL/kg distilled water, and 0.25 × 10^9^ CFU/kg *B. adolescentis*, respectively, by gavage for 21 days. **(A)** There was no significant difference in the Shannon index at the phylum level induced by *B. adolescentis*. **(B)** The principal component analysis (PCA) results showed no significant difference in the microbial community composition. **(C)** Community barplot analysis showed the community composition and species abundance in the three groups. **(D)** The decrease in *Firmicutes* abundance in the CRS group was enhanced by *B. adolescentis*. **(E)** The increased *Bacteroidetes* abundance in the CRS group was decreased by *B. adolescentis*. The data are shown as the mean ± SEM. One-way ANOVA followed by the Student-Newman-Keuls test was used. *p < 0.05 vs. the control; ^#^*p* < 0.05 vs. the CRS group; *n* = 3 per group. Con, Control; CRS, chronic restraint stress; Bif+CRS, *B. adolescentis* + chronic restraint stress.

**Figure 4:**
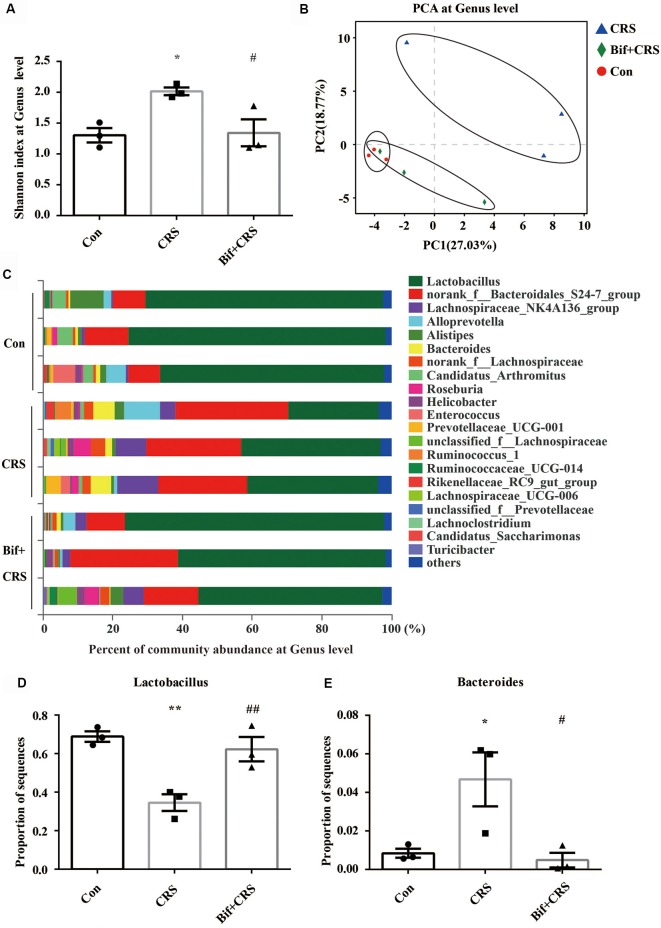
*B. adolescentis* reversed the imbalance of the intestinal microflora induced by CRS at the genus level. The mice in the Con, CRS, and Bif+CRS groups were treated with 10 mL/kg distilled water, 10 mL/kg distilled water, and 0.25 × 10^9^ CFU/kg *B. adolescentis*, respectively, by gavage for 21 days. **(A)** An increased Shannon index was observed in the CRS group compared with the control group, and the increase in the index was attenuated by *B. adolescentis*. **(B)** PCA revealed that the microbial community composition in the *B. adolescentis* group was more similar to that in the control than that in the CRS group, as shown by the clustering of the samples in the plots. **(C)** Community barplot analysis is shown. **(D)** The decrease in the *Lactobacillus* abundance in the CRS group was increased by *B. adolescentis*. **(E)** The enhanced *Bacteroides* abundance in the CRS group was reversed by *B. adolescentis*. The data are shown as the mean ± SEM. One-way ANOVA followed by the Student-Newman-Keuls test was used. *p < 0.05 and **p < 0.01 vs. the control; ^#^*p* < 0.05 and ^##^*p* < 0.01 vs. the CRS group; *n* = 3 per group. Con, Control; CRS, chronic restraint stress; Bif+CRS, *B. adolescentis* + chronic restraint stress.

**Figure 5:**
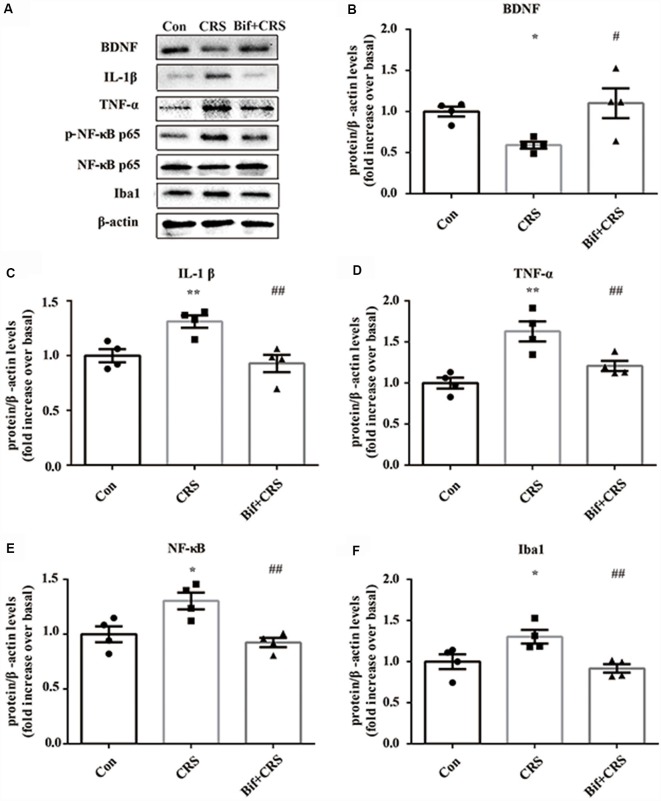
Changes in the protein expression of BDNF, IL-1β, TNF-α, p-NF-κB p65 and Iba1 in the hippocampus induced by pretreatment with *B. adolescentis*. The mice in the Con, CRS, and Bif+CRS groups were treated with 10 mL/kg distilled water, 10 mL/kg distilled water, and 0.25 × 10^9^ CFU/kg *B. adolescentis*, respectively, by gavage for 21 days. **(A)** Western blotting showed that **(B)** the protein level of BDNF was upregulated by *B. adolescentis*, and the protein levels of **(C)** IL-1β, **(D)** TNF-α, **(E)** p-NF-κB p65 and **(F)** Iba1 in the hippocampus were obviously suppressed by *B. adolescentis*. The data are shown as the mean ± SEM. One-way ANOVA followed by the Student-Newman-Keuls test was used. *p < 0.05 and **p < 0.01 vs. the control; ^#^*p* < 0.05 and ^##^*p* < 0.01 vs. the CRS group; *n* = 4 per group. Con, Control; CRS, chronic restraint stress; Bif+CRS, *B. adolescentis* + chronic restraint stress; BDNF, brain derived neurotrophic factor; IL-1β, interleukin-1β; TNF-α, tumor necrosis factor α; NF-κB, nuclear factor-kappa B; Iba1, ionized calcium binding adapter molecule 1.

**Figure 6:**
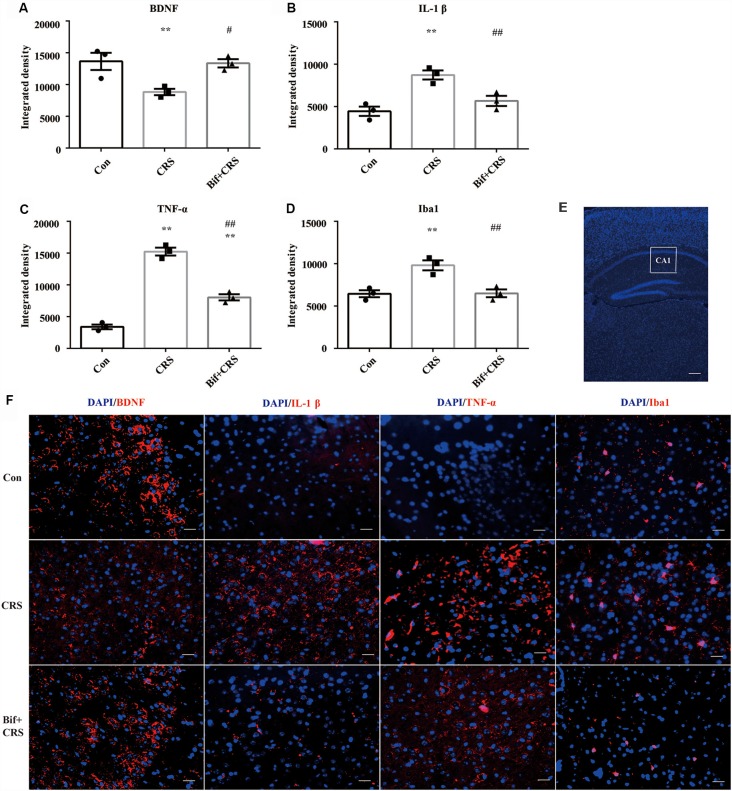
Changes in the protein expression of BDNF, IL-1β, TNF-α, and Iba1 in the CA1 region of the hippocampus induced by pretreatment with *B. adolescentis*. Immunofluorescence analysis showed that **(A)** the protein level of BDNF was upregulated by *B. adolescentis*, and the protein levels of **(B)** IL-1β, **(C)** TNF-α, and **(D)** Iba1 in the CA1 region of the hippocampus were obviously suppressed by *B. adolescentis*. The data are shown as the mean ± SEM. One-way ANOVA followed by the Student-Newman-Keuls test was used. **p < 0.01 vs. the control; ^#^*p* < 0.05 and ^##^*p* < 0.01 vs. the CRS group; *n* = 3 per group. **(E)** The CA1 region was identified as shown in the box. Scale bar = 200 μm. **(F)** Immunofluorescence was performed and subsequently visualized with Cy3 (red)-labeled secondary antibodies. DAPI (blue) was used as a nuclear stain. Scale bar = 20 μm. Con, Control; CRS, chronic restraint stress; Bif+CRS, *B. adolescentis* + chronic restraint stress; BDNF, brain derived neurotrophic factor; IL-1β, interleukin-1β; TNF-α, tumor necrosis factor α; Iba1, ionized calcium binding adapter molecule 1.

The mice were randomly allocated into three groups: the Con group, which received 10 mL/kg distilled water; the CRS group, which was subjected to the CRS procedure and received 10 mL/kg distilled water; and the Bif+CRS group, which was subjected to the CRS procedure and received 0.25 × 10^9^ CFU/kg *B. adolescentis*. Each group consisted of twelve mice. The mice were treated with distilled water or *B. adolescentis* by gavage for 21 days depending on the group. For the CRS procedure, the mice were placed in a 50-mL tube for 4 h for 21 consecutive days. The behavioral test procedure was performed as reported previously with few modifications (Yang et al., 2015). After 21 days, all mice underwent a behavioral test for four consecutive days (Figure 2). One day after the completion of the behavioral test, six mice from each group were anesthetized (6% sodium pentobarbital, intraperitoneally) and subsequently sacrificed by decapitation. The whole hippocampus was quickly dissected and frozen in liquid nitrogen and stored at −80°C for Western blotting (Figure 5). The cecal contents were collected and used for 16S rRNA sequence analysis (Figures 3, 4). After deep anesthesia with 6% sodium pentobarbital, six mice from each group were perfused with 4% paraformaldehyde for immunofluorescence labeling (Figure 6).

#### CRS

To establish the CRS model, the mice were placed in a horizontal resting position inside a well-ventilated (12 holes and 0.5 mm in diameter) 50-mL tube for 4 h for 21 consecutive days (Wong et al., 2016).

## Antibodies and Reagents

Rabbit anti-brain-derived neurotrophic factor (BDNF) antibody, rabbit anti-ionized calcium binding adapter molecule 1 (Iba1) antibody, and rabbit anti-IL-1β antibody were acquired from Abcam (Shanghai, China). Rabbit anti-TNF-α, rabbit anti-p-NFκBp65, rabbit anti-NFκBp65, β-actin, and horseradish peroxidase (HRP)-conjugated anti-rabbit IgG antibodies were obtained from Cell Signaling Technology (Boston, MA, USA). An E.Z.N.A. Stool DNA Kit was acquired from Omega Bio-Tek (Norcross, GA, USA). An AxyPrep DNA Gel Extraction Kit was obtained from Axygen Biosciences (Union City, CA, USA). Live *B. adolescentis* AY240946 was purchased from Lizhu Pharmaceutical Factory (Guangdong, China).

### Behavioral Tests

The illumination in the testing room was controlled, providing 60 lux inside the apparatus. Thirty minutes before the tests, each mouse was brought into the testing laboratory. The behavioral tests were carried out between 9:00 and 13:00.

#### Open Field Test (OFT)

The open field apparatus was a box (50 × 50 × 28 cm) with a floor divided into 25 squares. The nine central squares were defined as the center. On the day of the OFT, each mouse was gently placed in the center of the open field, and its movements were digitally recorded by video tracking software Smart V3.0 (Panlab, Spain) over a 6-min trial.

#### Elevated Plus-Maze (EPM) Test

On the day of the EPM test, each mouse was gently placed in the center of the EPM. The EPM was elevated 50 cm above the floor and consisted of two open arms (31 × 7.8 cm), two enclosed arms (31 × 6.5 × 11 cm) with walls, and a central area (7.8 × 6.5 cm). Movements were measured over a 6-min trial by Smart V3.0.

#### Tail Suspension Test (TST)

On the day of the tail suspension test, the mice were suspended upside down by their tails 40 cm above the floor, and an adhesive tape was placed 1 cm from the tail tip. The testing period was 6 min long, and the immobility time was analyzed over the last 4 min by Smart V3.0.

#### Forced Swimming Test (FST)

On the day of the FST, the mice were placed in a vertical transparent cylinder (30 cm in height and 12 cm in diameter) containing tap water at 25 ± 1°C and 20 cm in depth. The testing period was 6 min long, and the immobility time of each mouse was scored over the last 4 min by Smart V3.0.

### Analysis of Cecal Microflora Community Diversity

Previously described procedures (Ravel et al., 2011) were used. For 16S rRNA sequence analysis, DNA was extracted and then amplified to target the V1-V3 region with indexes and the adaptor-linked universal primers 27F (5′-AGAGTTTGATCCTGGCTCAG-3′) and 533R (5′-TTACCGCGGCTGCTGGCAC-3′). Amplicons were extracted, purified, and quantified by QuantiFluor-ST (Promega, San Luis Obispo, CA, USA). The purified amplicons were tagged with nucleotide barcodes, pooled in equimolar quantities and paired-end sequenced (2 × 300 bp) on an Illumina MiSeq platform. The raw fastq files were de-multiplexed, quality-filtered with Trimmomatic (Version 3.29), and merged with FLASH (version 1.2.7). Operational taxonomic units (OTUs) were collected with a 97% similarity cut-off by UPARSE (version 7.1^1^), and chimeric sequences were identified and removed by UCHIME. The taxonomy of each 16S rRNA gene sequence was analyzed by the RDP Classifier algorithm^2^ against the Silva (SSU128) 16S rRNA database with a confidence threshold of 70%. The data were analyzed on the free online platform called Majorbio I-Sanger Cloud Platform^3^.

### Western Blotting of the Hippocampus (Yuan et al., 2014)

Samples were homogenized with a protein extraction reagent containing protease inhibitors, and the concentrations of the proteins were determined using a BCA protein concentration assay kit. The proteins were separated by sodium dodecyl sulfate poly-acrylamide gel electrophoresis in a Mini-Protein II apparatus (Bio-Rad, CA, USA). The protein bands were electro-blotted onto a polyvinylidene fluoride membrane, and the membranes were blocked. The membranes were incubated with primary antibodies against BDNF (1:1,000), IL-1β (1:1,000), Iba1 (1:1,000), TNF-α (1:1,000), p-NF-κB p65 (1:1,000), and NF-κB p65 (1:1,000) overnight at 4°C. They were further incubated with a HRP-conjugated anti-rabbit IgG (1:5,000) antibody and then developed with ECL reagents. The different blots were incubated with different antibodies. As much of the proteins, which had different molecular weights, as possible were transferred to a single membrane, and then the blots were cut at different molecular weights and incubated with different antibodies according to the molecular weight of the target proteins. The target protein band was compared with the β-actin band on the same membrane. The chemiluminescence signal was imaged using a ChemiDoc XRS system (Bio-Rad) and the protein band signals were measured using ImageJ 1.4.3.67 software. The signal intensities of the individual protein bands were normalized to the β-actin band intensity and were represented by arbitrary units.

### Immunofluorescence Labeling in the Hippocampus (Fang et al., 2015)

The brains were removed and embedded in paraffin. Coronal sections of 7 μm thickness were cut using a microtome (Model: CUT5062; Mainz, SLEE, Germany), and the sections were blocked in 5% goat serum for 1 h at room temperature (22–24°C). After the serum was discarded, the sections were incubated with primary antibodies against BDNF (1:100), TNF-α (1:100), IL-1β (1:100), and Iba1 (1:100) overnight at 4°C. The sections were incubated with a Cy3-conjugated secondary antibody (1:200) for 1 h at room temperature and then mounted with a fluorescent mounting medium containing 4′,6-diamidino-2-phenylindole (DAPI). Eight successive slices from each mouse were used for immunofluorescence, and four successive slices (two arrays) were allocated for incubation with the different antibodies. The sections were subsequently observed by an inverted fluorescence microscope (Axio Observer. Z1, Zeiss, Germany). The CA1 region was assessed in the immunofluorescence and was represented on the template coronal slices from 1.70 mm to 1.94 mm inclusive, anterior to the bregma. The quantification of immunofluorescence intensity in the fluorescence images was expressed as integrated density, which was quantified using ImageJ software.

### Statistical Analysis

All data are presented as the mean ± standard error of mean (SEM). Statistical analyses were performed using a two-tailed Student’s *t*-test or one-way analysis of variance (ANOVA) followed by a Student-Newman-Keuls *post hoc* test using SPSS 19.0 (SPSS Inc., Chicago, IL, USA). For the microbiota, the Adonis test was used to analyze the ß diversity in principal component analysis (PCA), and False Discovery Rate (FDR) was used to correct for multiple testing of the comparisons of specific genera. A *p*-value < 0.05 was considered statistically significant.

## Results

### Potential Anxiolytic and Antidepressant Effects of *B. adolescentis*

To preliminarily investigate the antidepressant effects of *B. adolescentis*, the FST was used. The immobility duration (*t*_18_ = 2.698, *p* = 0.015; *t*_18_ = 2.741, *p* = 0.013; *t*_18_ = 2.794, *p* = 0.012; *t*_18_ = 2.824, *p* = 0.011; Figure 1B) was decreased significantly by amitriptyline (10 mg/kg) and *B. adolescentis* (0.25 × 10^9^ CFU/kg, 0.5 × 10^9^ CFU/kg, and 1 × 10^9^ CFU/kg). The OFT and the EPM were used to evaluate spontaneous activity and the anxiolytic effects of *B. adolescentis*. The total distance traveled in the OFT (*t*_18_ = −0.995, *p* = 0.333, Figure 1C) did not significantly differ between the control group and the *B. adolescentis*-treated (0.25 × 10^9^ CFU/kg) group, and the time spent in the center of the OFT apparatus (*t*_18_ = −3.11, *p* = 0.006, Figure 1D) was increased by *B. adolescentis* (0.25 × 10^9^ CFU/kg). Moreover, the total number of entries into the arms of the EPM (*t*_18_ = −1.087, *p* = 0.292, Figure 1E) did not differ between the control group and the *B. adolescentis*-treated (0.25 × 10^9^ CFU/kg) group. *B. adolescentis* (0.25 × 10^9^ CFU/kg) increased the percentage of entries into the open arms of the EPM (*t*_18_ = −2.768, *p* = 0.013, Figure 1F). However, *B. adolescentis* (0.25 × 10^9^ CFU/kg) did not increase the percentage of time spent in the open arms of EPM (*t*_18_ = −2.018, *p* = 0.059, Figure 1G). These data (Table 1) indicate the potential anxiolytic and antidepressant effects of *B. adolescentis*.

**Table 1:** Potential anxiolytic and antidepressant effects of *B. adolescentis*.

	B. Immobility Duration of Forced swimming test (%)				C. Total Distance of Open Field (cm)		D. Time in Center of Open Field (%)		E. Total Entries of EPM		F. Entries in Open Arms of EPM (%)		G. Time in Open Arms of EPM (%)	
Con	Ami (10 mg/kg)	Bif (0.25×10^9^ CFU/kg)	Bif (0.5×10^9^ CFU/kg)	Bif (1×10^9^ CFU/kg)	Con	Bif (0.25×10^9^ CFU/kg)	Con	Bif (0.25×10^9^ CFU/kg)	Con	Bif (0.25×10^9^ CFU/kg)	Con	Bif (0.25×10^9^ CFU/kg)	Con	Bif (0.25×10^9^ CFU/kg)
39.17	2.5	28.89	22.22	16.69	1263.21	2053.69	4.43	7.34	15	10	26.67	20.00	32.24	26.28
27.78	4.44	23.17	12.5	14.81	1595.11	2465.83	4.67	12.69	14	8	14.29	37.50	14.92	35.56
21.94	23.06	11.11	29.72	17.08	1121.71	1635.51	2.51	9.32	16	26	12.50	34.62	18.52	62.16
21.39	3.06	22.08	7.22	13.97	1242.33	2493.29	2.44	12.36	8	18	12.50	16.67	33.28	34.88
30.56	10.56	6.39	25	12.67	1382.98	504.9	5.37	8.56	19	12	0.00	41.67	0	54.24
23.06	31.11	25.83	13.06	30.67	1480.85	971.53	9.64	4.28	18	15	11.11	40.00	10.24	31.48
24.44	14.17	12.78	23.33	15.47	1577.51	1446.7	0.45	6.13	7	22	14.29	18.18	26.4	42.32
51.11	18.06	20.28	15.83	20.27	972.73	2119.14	3.35	4.21	9	27	33.33	25.93	42.52	32.76
23.06	35	18.06	18.61	26.53	2412.68	1785.53	6.4	14.25	22	15	13.64	20.00	14.72	28
27.22	21.11	19.72	20	22.42	1324.97	1272.91	4.65	7.2	12	15	25.00	26.67	48.6	20.52
compared with control *t*-test for Equality of Means					compared with control		compared with control		compared with control		compared with control		compared with control	
					*t*-test for Equality of Means		*t*-test for Equality of Means		*t*-test for Equality of Means		*t*-test for Equality of Means		*t*-test for Equality of Means	
*p* = 0.015		0.013	0.012	0.011	*p* = 0.333		*p* = 0.006		0.292		0.013		0.059	
*df* = 18		18	18	18	*df* = 18		*df* = 18		*df* = 18		*df* = 18		*df* = 18	
*t* = 2.698		2.741	2.794	2.824	*t* = −0.995		*t* = −3.11		*t* = −1.087		*t* = −2.768		*t* = −2.018	

*The mice in groups of Con, Ami (10 mg/kg), Bif(0.25×10^9^ CFU/kg), Bif (0.5×10^9^ CFU/kg) and Bif (1×10^9^ CFU/kg) were given with distilled water (10 mL/kg), amitriptyline (10 mg/kg), *B. adolescentis* (0.25×10^9^ CFU/kg), *B. adolescentis* (0.5×10^9^ CFU/kg) and *B. adolescentis* (1×10^9^ CFU/kg) respectively, by gavage for 21 days. N = 10 per group*.

### Anxiolytic and Antidepressant Effects of *B. adolescentis* in CRS Mice

To further investigate the anxiolytic and antidepressant effects of *B. adolescentis*, a CRS model was used, as shown in the schedule (Figure 2A). There was a significant difference in the time spent in the center of the OFT apparatus (*F*_(2,33)_ = 4.183, *p* = 0.024, Figure 2B). The time spent in the center of the OFT apparatus was decreased in the CRS group compared with the control group (*p* < 0.05), and the *B. adolescentis* group spent a significantly longer time in the center than that spent by the CRS group (*p* < 0.05). There were no remarkable changes among the three groups in the total distance traveled in the OFT (*F*_(2,33)_ = 1.875, *p* = 0.169, Figure 2C). The EPM test showed no significant difference between the two groups in the total number of entries (*F*_(2,33)_ = 0.104, *p* = 0.901, Figure 2D). *B. adolescentis* increased the percentage of entries into the open arms of the EPM (*F*_(2,33)_ = 9.108, *p* = 0.001, Figure 2E) and the percentage of time spent in the open arms of the EPM (*F*_(2,33)_ = 3.465, *p* = 0.043, Figure 2F). Both the percentage of entries into the open arms of the EPM (*p* < 0.01) and the percentage of time spent in the open arms of the EPM (*p* < 0.05) were decreased in the CRS group compared with the control group. Both percentages (*p* < 0.01, *p* < 0.05) were increased in the *B. adolescentis* group compared with the CRS group. There was a significant difference in the immobility durations in the TST (*F*_(2,33)_ = 3.422, *p* = 0.045, Figure 2G) and the FST (*F*_(2,33)_ = 5.78, *p* = 0.007, Figure 2H). The results showed markedly increased immobility durations in the CRS mice in both the TST (*p* < 0.05) and the FST (*p* < 0.01). Decreased immobility durations were observed in both the TST (*p* < 0.05) and the FST (*p* < 0.01) in the *B. adolescentis* group compared with the CRS group. Altogether, these data (Table 2) indicate that *B. adolescentis* has anxiolytic and antidepressant effects on CRS mice in these behavioral tests.

**Table 2:** Anxiolytic and antidepressant effects of *B. adolescentis* in CRS mice.

B. Time in Center of Open Field (%)			C. Total Distance of Open Field (cm)			D. Total Entries of EPM			E. Entries in Open Arms of EPM (%)			F. Time in Open Arms of EPM (%)			G. Immobility Duration of Tail Suspension Test (%)			H. Immobility Duration of Forced swimming test (%)		
Con	CRS	Bif+CRS	Con	CRS	Bif+CRS	Con	CRS	Bif+CRS	Con	CRS	Bif+CRS	Con	CRS	Bif+CRS	Con	CRS	Bif+CRS	Con	CRS	Bif+CRS
5.39	0.32	2.6	1941.64	1658.32	1543.94	15	13	8	20.00	15.38	25.00	21.32	16.32	23.17	22.05	34.53	11.55	5.9	19.33	15.18
5.81	2.84	12.29	1977.99	1462.89	2161.88	19	21	14	26.32	14.29	28.57	25.44	24.48	36.54	2.82	42.82	12.15	8.31	15.45	2.22
8.02	3.78	1.7	2243.67	1696.25	2026.44	23	5	6	21.74	0.00	16.67	23.58	0	17.25	1.35	24.54	32.22	22	29.35	14.88
6.96	0.56	6.97	1854.72	1604.73	2330.81	10	8	20	20.00	12.50	20.00	21.95	15.12	18.94	14.9	22.68	17.27	6.83	35.9	1.03
7.01	3.02	5.51	1936.98	2234.28	2407.91	25	24	10	16.00	16.67	20.00	28.33	18.29	23.19	5.25	22.4	0	9.88	10.32	7.53
4.51	3.76	10.56	2572.29	1902.54	1907.05	6	15	9	16.67	6.67	33.33	22.03	10.54	34	24.03	14.9	12.77	9.62	22.03	3.63
2.46	1.46	4.62	1704.71	1608.22	2393.15	21	10	30	28.57	20.00	23.33	34.26	29.98	20.05	11.58	13.92	13.02	19.22	7.22	24.97
3.71	4.98	6.31	1965.25	1994.96	1623.36	8	22	22	12.50	13.64	9.09	9.35	14.76	5.27	13.52	11.15	6.68	0.62	18.73	7.67
16.11	3.96	2.26	2274.03	1956.94	2101.34	17	11	19	17.65	9.09	15.79	29.42	10	15.74	0.4	33.27	19.03	1.05	7.52	5.53
4.64	3.96	12.06	1529.13	1588.10	2308.45	10	18	27	10.00	11.11	18.52	4.54	11.85	19.57	5	21.32	18.25	7.03	15.78	1.7
5.6	6.53	13.08	2111.62	2502.09	2453.90	20	12	17	25.00	8.33	23.53	26	7.42	22.46	35.6	13.92	3.95	12.27	18.25	21.28
10.18	3.51	2.25	2385.22	1873.10	1532.85	18	19	8	16.67	15.79	25.00	17.55	14.11	26.57	23.95	12.72	9.65	8.64	20.33	3.08
*df*	*F*	*P*	*df*	*F*	*P*	*df*	*F*	*P*	*df*	*F*	*P*	*df*	*F*	*P*	*df*	*F*	*P*	*df*	*F*	*P*
2,33	4.183	0.024	2,33	1.875	0.169	2,33	0.104	0.901	2,33	9.108	0.001	2,33	3.465	0.043	2,33	3.422	0.045	2,33	5.78	0.007

*The mice in groups of Con, CRS and Bif+CRS were given with distilled water (10 mL/kg), distilled water (10 mL/kg), *B. adolescentis* (0.25×10^9^ CFU/kg) respectively, by gavage for 21 days. N = 12 per group*.

### *B. adolescentis* Reversed the Imbalance of Cecal Microflora Induced by CRS

To test the assumption that the anxiolytic and antidepressant effects of *B. adolescentis* are related to rebalancing the gut microbiota, cecal microflora community diversity was analyzed. There was no significant difference in the Shannon index at the phylum level (*F*_(2,6)_ = 4.5, *p* = 0.064, Figure 3A). PCA revealed that there was no significant difference in the microbial community composition of the three groups at the phylum level (*R*^2^ = 0.53264, *p* = 0.108, Figure 3B). Community barplot analysis showed the community composition and species abundance in the three groups (Figure 3C). Further analysis showed a significant difference in the abundance of *Firmicutes* (*F*_(2,6)_ = 5.946, *p* = 0.038, Figure 3D) and *Bacteroidetes* (*F*_(2,6)_ = 5.9, *p* = 0.038, Figure 3E) at the phylum level. The decline (*p* < 0.05) in *Firmicutes* abundance in the CRS group was enhanced (*p* < 0.05) by *B. adolescentis*. Additionally, the previous increase (*p* < 0.05) in *Bacteroidetes* in the CRS group was decreased (*p* < 0.05) by *B. adolescentis* (Table 3).

**Table 3:** *B. adolescentis* reversed the imbalance of the intestinal microflora induced by CRS at the phylum level.

A. Shannon index on Phylum level			B. PCA on Phylum level			D. Proportion of sequences on Phylum level: Firmicutes			E. Proportion of sequences on Phylum level: Bacteroidetes		
Con	CRS	Bif+CRS		PC1	PC2	Con	CRS	Bif+CRS	Con	CRS	Bif+CRS
0.618248	0.804381	0.538529	Con	1.4023	−0.1719	0.7599	0.4212	0.8048	0.2271	0.5547	0.186
0.490792	0.785661	0.652056		1.4472	0.1895	0.8335	0.646	0.6492	0.1586	0.3226	0.3261
0.639374	0.737641	0.754216		0.7645	2.1739	0.7751	0.6067	0.7693	0.1977	0.3805	0.2002
			CRS	−1.253	−1.4395						
*df*	*F*	*P*		−3.2233	1.7086	*df*	*F*	*P*	*df*	*F*	*P*
2,6	4.5	0.064		−0.8385	−2.0185	2,6	5.946	0.038	2,6	5.9	0.038
			Bif+CRS	1.5016	−0.047						
				−0.1986	−0.9116						
				0.3979	0.5164						
			*R*^2^ = 0.53264, *p* = 0.108								

*The mice in groups of Con, CRS and Bif+CRS were given with distilled water (10 mL/kg), distilled water (10 mL/kg), B. adolescentis (0.25×109 CFU/kg) respectively, by gavage for 21 days. N = 3 per group*.

For the further verification of cecal microflora community diversity, Shannon index, principal component and community barplot analyses were performed at the genus level. There was a significant difference in the Shannon index at the genus level (*F*_(2,6)_ = 7.294, *p* = 0.025, Figure 4A). An increased Shannon index (*p* < 0.05) was observed in the CRS group compared with the control group, and the increase in the index was attenuated (*p* < 0.05) by *B. adolescentis*. PCA revealed that the microbial community composition in the *B. adolescentis* group was more similar to that in the control group than that in the CRS group, as shown by the clustering of the samples in the plots (*R*^2^ = 0.54811, *p* = 0.008, Figure 4B). Community barplot analysis is shown in Figure 4C. Significant differences in the abundance of *Lactobacillus* (*F*_(2,6)_ = 14.997, *p* = 0.005, Figure 4D) and *Bacteroides* (*F*_(2,6)_ = 7.488, *p* = 0.023, Figure 4E) are shown. The decrease in *Lactobacillus* abundance (*p* < 0.01) in the CRS group was increased (*p* < 0.01) by *B. adolescentis*. Furthermore, the enhanced abundance of *Bacteroides* (*p* < 0.05) in the CRS group was also reversed (*p* < 0.05) by *B. adolescentis* (Table 4). The 16S rRNA sequencing datasets for this study were deposited at NCBI and are accessible at https://www.ncbi.nlm.nih.gov/sra/PRJNA498761.

**Table 4:** *B. adolescentis* reversed the imbalance of the intestinal microflora induced by CRS at the genus level.

A. Shannon index on Genus level			B. PCA on Genus level			D. Proportion of sequences on Genus level: Lactobacillus			E. Proportion of sequences on Genus level: Bacteroides		
Con	CRS	Bif+CRS		NMDS1	NMDS2	Con	CRS	Bif+CRS	Con	CRS	Bif+CRS
1.29678	2.136918	1.103383	Con	−4.3868	−1.0162	0.684	0.261	0.7448	0.0057	0.0618	0.0125
1.106301	1.925281	1.781085		−3.218	−1.3881	0.7375	0.4001	0.5294	0.0065	0.0188	0.0016
1.511226	1.984399	1.145668		−4.0307	−0.46	0.6449	0.3768	0.5958	0.0131	0.0598	0.0006
			CRS	−1.8479	9.6013						
*df*	*F*	*P*		7.3032	−0.9993	*df*	*F*	*P*	*df*	*F*	*P*
2,6	7.294	0.025		8.495	2.9059	2,6	14.997	0.005	2,6	7.488	0.023
			Bif+CRS	−3.6345	−0.6355						
				−2.0275	−2.618						
				3.3472	−5.3902						
			*R*^2^ = 0.54811, *p* = 0.008								

*The mice in groups of Con, CRS and Bif+CRS were given with distilled water (10 mL/kg), distilled water (10 mL/kg), B. adolescentis (0.25×109 CFU/kg) respectively, by gavage for 21 days. N = 3 per group*.

### *B. adolescentis* Increased BDNF Expression and Reduced the Expression of Inflammatory Cytokines in the Hippocampus of CRS Mice

To investigate the relationship between the anti-inflammatory effects and the anxiolytic/antidepressant effects of *B. adolescentis* and to verify the effects of the inflammatory conditions that are induced by chronic stress, we evaluated protein expression in the hippocampus, which is related to inflammation and chronic stress (Sathyanesan et al., 2017). Chronic stress can trigger both anxiety- and depression-like behaviors as well as reduce BDNF levels (Berry et al., 2012) and additionally, cytokines can attenuate the BDNF level in depression (Yu and Chen, 2011). There was a significant difference in BDNF expression (*F*_(2,9)_ = 5.731, *p* = 0.025, Figure 5B). It is striking that the decreased BDNF expression (*p* < 0.05) in the CRS treated with *B. adolescentis* showed the most drastic increase (*p* < 0.05). Western blotting analysis also showed that the protein levels of IL-1β (*F*_(2,9)_ = 9.477, *p* = 0.006, Figure 5C), TNF-α (*F*_(2,9)_ = 13.469, *p* = 0.002, Figure 5D), p-NF-κB p65 (*F*_(2,9)_ = 9.3, *p* = 0.006, Figure 5E) and Iba1 (*F*_(2,9)_ = 7.066, *p* = 0.014, Figure 5F) in the hippocampus were significantly different. The increases (*p* < 0.01, *p* < 0.01, *p* < 0.05, *p* < 0.05) were clearly suppressed (*p* < 0.01, *p* < 0.01, *p* < 0.01, *p* < 0.01) in the *B. adolescentis*-treated mice compared with the CRS mice (Table 5). To further explore the correlation between anxiety/depression and inflammation, the protein expression of BDNF, IL-1β, TNF-α and Iba1 were examined in the CA1 region (Berkiks et al., 2018) of the hippocampus, as shown in Figure 6E. The protein expression of BDNF (*F*_(2,6)_ = 8.868, *p* = 0.016, Figure 6A), IL-1β (*F*_(2,6)_ = 15.471, *p* = 0.004, Figure 6B), TNF-α (*F*_(2,6)_ = 143.837, *p* = 0, Figure 6C) and Iba1 (*F*_(2,6)_ = 15.24, *p* = 0.004, Figure 6D) was verified by immunofluorescence (Figure 6F). The integrated density results showed that decreased BDNF expression (*p* < 0.01) was increased by *B. adolescentis* (*p* < 0.05), and the enhanced expression of IL-1β (*p* < 0.01), TNF-α (*p* < 0.01) and Iba1 (*p* < 0.01) was reduced (*p* < 0.01, *p* < 0.01, *p* < 0.01) by *B. adolescentis* (Table 6). The results imply that *B. adolescentis* increases BDNF expression under CRS conditions related to its anti-inflammatory effects in the hippocampus.

**Table 5:** Changes in the protein expression of BDNF, IL-1β, TNF-α, p-NF-κB p65 and Iba1 in the hippocampus induced by pretreatment with *B. adolescentis*.

	B. Western blotting analysis for BDNF, IL-1β, TNF-α, p-NF-κ B p65 and Iba1 expression				
	BDNF	IL-1β	TNF-α	p-NF-κB p65/NF-κB p65	Iba1
Con	1.0723	0.9218	0.9743	0.9441	1.1421
	1.0099	1.1345	0.8306	0.8215	0.7453
	1.0872	0.8812	1.0628	1.1499	1.1128
	0.8307	1.0634	1.1325	1.0852	1.0011
CRS	0.4894	1.1463	1.9127	1.1227	1.1859
	0.6007	1.3988	1.7253	1.2378	1.1808
	0.6971	1.3309	1.3475	1.4009	1.3169
	0.5803	1.3691	1.5323	1.4565	1.5296
Bif+CRS	1.5287	0.6976	1.1223	0.9601	0.8247
	1.1179	0.9694	1.3875	0.8072	1.0179
	1.1195	1.0616	1.1956	0.9221	0.9956
	0.6419	0.9869	1.1328	1.0082	0.8336
*df*	2,9	2,9	2,9	2,9	2,9
*F*	5.731	9.477	13.469	9.3	7.066
*P*	0.025	0.006	0.002	0.006	0.014

*The mice in groups of Con, CRS and Bif+CRS were given with distilled water (10 mL/kg), distilled water (10 mL/kg), B. adolescentis (0.25×109 CFU/kg) respectively, by gavage for 21 days. N = 4 per group*.

**Table 6:** Changes in the protein expression of BDNF, IL-1β, TNF-α and Iba1 in the CA1 region of the hippocampus induced by pretreatment with *B. adolescentis*.

	Con			CRS			Bif+CRS			*df*	*F*	*P*
BDNF	14,751	10,982	15,237	8,019	9,727	8,762	13,296	14,505	12,274	2,6	8.868	0.016
IL-1β	3,433	4,576	5,334	8,892	7,734	9,565	4,654	5,672	6,708	2,6	15.471	0.004
TNF-α	2,829	4,075	3,323	15,288	14,169	16,283	7,956	8,932	7,249	2,6	143.837	0
Iba1	6,533	7,142	5,706	10,026	8,717	10,696	5,719	6,477	7,329	2,6	15.24	0.004

*The mice in groups of Con, CRS and Bif+CRS were given with distilled water (10 mL/kg), distilled water (10 mL/kg), B. adolescentis (0.25 × 109 CFU/kg), respectively, by gavage for 21 days. N = 3 per group*.

## Discussion

Here, we present novel evidence that *B. adolescentis* prevents the development of anxiety- and depression-like behaviors caused by CRS and that the effects of *B. adolescentis* are related to reducing inflammatory cytokines and rebalancing the gut microbiota.

The gut immune system consists of 70%–80% of the body’s immune cells (Furness et al., 1999). Injury to the gastrointestinal tract, which is induced by CRS and related to the alteration of intestinal flora, triggers the discrimination of pathogenic and commensal microorganisms by the gut immune system and an immune response to the pathogen (Artis, 2008). The immune system is an indispensable part of central nervous system activity, including anxiety (Bercik et al., 2010) and depression (Hayley, 2011; Park et al., 2011). Additionally, intestinal flora exert substantial impacts on brain function through immune pathways by inducing the release of inflammatory cytokines into the circulatory system and then into the brain through the transport system of the blood brain barrier to directly influence brain activity and function (Pfau et al., 2018). Moreover, alterations in brain activity and function contribute to behavioral changes (Foster, 2016).

Depressed patients exhibit dysbiosis of gut bacteria or alterations in enteric microorganisms (Aizawa et al., 2016; Zheng et al., 2016; Dinan and Cryan, 2017; Fung et al., 2017). Chronic stress and depression were found to increase both the diversity and richness of gut bacterial populations (Naseribafrouei et al., 2014; Jiang et al., 2015; Li et al., 2018), but this result is in contrast to others (Kelly et al., 2016; Bharwani et al., 2017). In our study, an increase in gut bacterial α-diversity (Shannon index) was found in the CRS group, indicating that it is possible that CRS triggers an increase in the diversity of microbiota similar to dysbiosis induced by depression in patients.

Furthermore, *Lactobacillus* was reduced in the CRS group compared with the control group. Women who receive *Lactobacillus rhamnosus* HN001 have significantly lower depression and anxiety scores in the postpartum period, and appropriate intervention with *Lactobacillus* is beneficial for depressive patients (Slykerman et al., 2017). Our data reinforce the essential link between anxiety/depression and the abundance of *Lactobacillus*. The data showed an increase in *Lactobacillus* in the CRS plus *B. adolescentis* group, verifying that the *Lactobacillus* increase was probably a major factor in preventing anxiety and depression. Consistent with the previous study, this study presumes that highly diverse bacterial communities, which are likely included in the pathogenesis of stress-induced behavioral deficits, are related to decreased *Lactobacillus* abundance (Li et al., 2018). The recovery of intestinal *Lactobacillus* levels is effective in improving behavioral deficits. Host kynurenine metabolism may be suppressed by reactive oxygen species derived by *Lactobacillus via* inhibiting the metabolizing enzyme IDO1 in the intestine (Marin et al., 2017).

It has been reported that 80% of patients with depression have a high proportion of *Bacteroides* in the fecal microbiota (Liu et al., 2016). As our study showed, the increased *Bacteroides* proportion in the CRS group was reversed by pretreatment with *B. adolescentis*, and at the same time, the decreased *Firmicutes* proportion in the CRS group was reversed by pretreatment with *B. adolescentis*. However, another controversial report showed that (R)-ketamine significantly increases the levels of Bacteroidales in susceptible mice after chronic social defeat stress (CSDS; Qu et al., 2017). The difference between the trends of Bacteroidales and *Bacteroides* may result from the different models, drugs and drug doses. The antidepressant effects of (R)-ketamine in a CSDS model may be regulated by the rebalancing of the intestinal microbiota to some extent, and (R)-ketamine can reduce the level of *Butyricimonas* in susceptible mice (Yang et al., 2017b). Moreover, *Clostridium butyricum*, a probiotic, augments 5-HT and BDNF expression, and its antidepressant effects in mice exposed to chronic unpredictable mild stress are partly due to the stimulation of intestinal glucagon-like peptide-1 secretion (Sun et al., 2018). The distinct appearance of *Bifidobacterium* is discovered in unsusceptible (resilient) mice and additionally, the administration of *Bifidobacterium* may confer resilience against CSDS (Yang et al., 2017a). Together, these findings show that the antidepressant mechanisms of *Bifidobacterium* related to the intestinal flora are complicated and require further investigation.

Gut microbiota can influence peripheral inflammation or CNS processes and subsequent behavioral responses to stress through cytokine-mediated immune signaling pathways, such as by increasing TNF-α (Ren et al., 2018), IL-1β (Grenham et al., 2011), and NF-κB (Wang et al., 2016), which is consistent with our data. Gut microbiota analysis shows that an anti-mouse IL-6 receptor antibody significantly improves the decreased *Firmicutes*/*Bacteroidetes* ratio in susceptible mice in a CSDS model (Zhang et al., 2017). It is possible that the increased *Firmicutes* and decreased *Bacteroidetes* levels induced by *B. adolescentis* are related to blockade of the IL-6 receptor. The gene expression of IL-1β in bone marrow-derived macrophage cells is increased after treatment with an isolate of *Bacteroides fragilis* (Deng et al., 2016). The bacterial flora of neonatal necrotizing enterocolitis patients contain significantly higher amounts of *Bacteroides*, which is accompanied by an enhancement of TNF-α (Hui et al., 2017), and *Bacteroides vulgatus* induces NF-κB activation and pro-inflammatory gene expression in intestinal epithelial cells (Haller et al., 2003). These studies show that *Bacteroides* is positively correlated with inflammatory factors, including IL-1β, TNF-α, and NF-κB. Additionally, a study demonstrated that the anxiolytic effect of *B. adolescentis* IM38 in mice may occur *via* reducing the blood IL-6 and TNF-α levels (Jang et al., 2018). Our data also showed an increase in *Bacteroides* and inflammatory cytokines, including IL-1β, TNF-α and NF-κB, in the CRS group, and the downregulation of these cytokines after pretreatment with *B. adolescentis*, confirming that a decreased *Bacteroides* level may be a vital element in the anti-inflammatory effects of *B. adolescentis*.

*Lactobacillus brevis* 23017 relieves colon toxicity by modulating oxidative stress and inflammation through NF-κB signaling cascades (Jiang et al., 2018). Additionally, our data demonstrate a reduction in *Lactobacillus* in the CRS group and the upregulation of *Lactobacillus* after pretreatment with *B. adolescentis*, verifying that an increase in *Lactobacillus* may be another key factor in the anti-inflammatory actions of *B. adolescentis*.

Microglia, which participate in synaptic pruning, promoting tissue repair, and recruiting peripheral leukocytes to sites of inflammation, have been observed in brain regions such as the hippocampus, amygdala, and prefrontal cortex (Wohleb, 2016; Ménard et al., 2017). *B. adolescentis* NK98 can inhibit the infiltration of Iba1 into the hippocampus caused by the acute immobilization stress (Jang et al., 2019). In our study, the *B. adolescentis*-pretreated group showed reduced Iba1 levels following CRS, which is in agreement with previously published studies that have highlighted the potential therapeutic efficacy of targeting central inflammatory processes, particularly those mediated by microglia, in depression (Alcocer-Gómez et al., 2016).

This study has a few limitations. First, in the preliminary efficacy screening experiment, *B. adolescentis* showed anxiolytic and antidepressant effects, then the mice were divided into a control group and a *B. adolescentis*-treated group. In our subsequent experiment, to study the anxiolytic and antidepressant mechanisms of *B. adolescentis*, we established a *B. adolescentis* treatment plus CRS group, but we did not use a *B. adolescentis*-treated group as a control. Second, our current analyses of the gut microbiota and the brain were obtained from three or four samples per group; therefore, a large sample size may be needed in future studies to improve our understanding of the mechanisms underlying the relationship between the gut microbiota and inflammation. Third, the current study is correlational in nature, so no conclusions can be drawn about whether the inflammatory effects are the mechanism for the treatment effect on behavior. It could equally be the case that there is some other mechanism and the inflammatory findings are merely tangential.

## Conclusion

In conclusion, we found that *B. adolescentis* increases the sequence proportion of *Lactobacillus* and reduces the sequence proportion of *Bacteroides* in feces. Moreover, *B. adolescentis* decreases IL-1β, TNF-α, NF-κB, and Iba1 protein expression and increases BDNF protein expression in the hippocampus of CRS mice. *B. adolescentis* has anxiolytic and antidepressant effects on behavioral performance in mice exposed to CRS. Thus, we conclude that the anxiolytic and antidepressant effects of *B. adolescentis* are related to reducing inflammatory cytokines and rebalancing the gut microbiota. Our data will contribute to the understanding of anti-inflammatory effects and the rebalancing of the gut microbiota as possible essential experimental therapeutic strategies for anxiety and depression.

## Ethics Statement

Male ICR mice were purchased from Kunming Medical University. The procedures were approved by the Institutional Animal Care and Use Committee of Kunming Medical University, and were performed in accordance with the Guide for the Care and Use of Laboratory Animals.

## Author Contributions

YG and J-PX: conceptualization. KD, X-MH, QW and J-JL: data curation. XL, YY, QX and JX: formal analysis. YG, YY and QW: funding acquisition. H-RL: methodology and writing—review and editing. YG: writing—original draft.

## Conflict of Interest Statement

The authors declare that the research was conducted in the absence of any commercial or financial relationships that could be construed as a potential conflict of interest.

## Footnotes

^1^http://drive5.com/uparse/

^2^http://rdp.cme.msu.edu/

^3^www.i-sanger.com
